# The Abuse of Exercise

**Published:** 1900-06-23

**Authors:** Thomas D. Lister

**Affiliations:** Pathologist to the East London Hospital for Children; Assistant Medical Officer to the Royal Exchange Assurance Company.


### Hospital Clinics and Medical Progress.

### THE ABUSE OF EXERCISE.

By Thomas D. Lister, M.D.Lond., F.R.C.S., Pathologist to the East London Hospital for Children; Assistant Medical Officer to the Royal Exchange Assurance Company.

There is a common tendency among a number of city-dwellers nowadays to take but little exercise during the winter months. The short days, the long evenings, the uncertain weather, the pressure of business due to the closing of the year's accounts, in fact, general circumstances combine to lessen the amount of outdoor exercise taken at that time. With young people the same tendency exists owing to the common educational systems. It is not, perhaps, altogether an unwholesome movement which causes the long grey months to work for the reward of a short summer of pleasure. But it is one that, once begun, must inevitably gain force with the growing demands made on energies, knowledge, and intelligence in the working months by the increasingly-rapid inter-communications and severe competition of advancing civilisation. It is also, therefore, one which makes for increased arduousness in the forms of relaxation which are enjoyed in the playing hours, and leads quite frequently to an abuse of otherwise healthful exercise.

As a result of what is possibly an enforced neglect of personal muscular activity for a large portion of the year, there is a considerable class to whom exercise is a disagreeable sort of medicine which is taken in holiday doses only. This dose-taking of exercise is particularly pernicious unless the dose is estimated as carefully as that of the heart poisons, and medically regulated for individual cases. Unfortunately, the simple direction, "Don't overdo it," is usually all that the patient hears, and, like the powder that is to be measured by the amount that will lie on the point of a knife, exercise is taken very much at the patient's discretion, or indiscretion, as the case may be. Few are taught to appreciate the symptoms of approach to the limit where the harmless merges into the harmful, and many mistake the immediate attempts of the body to compensate for overstrain for the actual establishment of new abilities.

Every physician's case-book furnishes examples of the abuse of exercise. They are not by any means limited in their occurrence to those only who indulge in irregular outbursts of muscular energy. Indeed, the most typical cases are inevitably seen, as with abuse of alcohol, among those continuously addicted to excess. But, also as with alcohol, unaccustomed indulgence may be followed by temporary or permanent damage of a kind and degree that varies with the relative excess and physiological resiliency of the individual at the time. And, as a consequence of the over-taxing of the nervous, vascular, and muscular systems in the holiday season, people are often anything but the better for the summer change, and prolonged exhaustion often follows the fine week-end outing among those employed in the office, warehouse, and shop.

The not at all infrequently-heard expression of gladness on coming back after the holidays has a good deal of its origin, especially among business men, in the outcry of an unrecognised but helpful subliminal consciousness. It marks the craving of the body for a return to normal living after an orgie of fatigue endured during a "rest and change of air." It would seldom be used in the sense here indicated if these people looked after their muscular condition in the intervals of their business, or if they left the special holiday exertions to those who had so qualified themselves to enjoy them. One class of society may be particularised, however, as furnishing but rare instances of the abuse of exercise. It is seldom seen among matrons, partly because they carry a good deal of their regular routine into their holiday life, especially if accompanied by their families, and partly because they have a more sensitive instinct than men to guard them against violent efforts, or, as they express it in feminine phrase, they have more sense. From their example a golden rule for the safety and enjoyment of a summer holiday is readily deducible for the non-athletic, and one may venture to phrase it thus: "Unless properly trained, live the physical life much as usual when away from home, revel in the rest and air and light, and, above all, do not eat more than you generally do when in health."

Considering the profound and universal effects of muscular activity on the functions and metabolism of the body, so deeply studied by physiologists, so widely known among physicians, effects which may be beneficial or hurtful according to circumstances, it is almost surprising to find that it frequently receives less careful medical regulation than diet and clothing. Few would now be found to dispute the assertion that exercise of the right kind and amount is as important to the attainment of perfect bodily health as food of the right quality and quantity. It may be said to be almost as often wrongly estimated. More people die of overfeeding than of overfasting, and, it is also true, that, excluding overfeeding from the habits of the non-athletic, more of such people shorten their lives by the abuse of exercise than by taking none at all except their daily journeyings. The increasing prevalence of cases arising from the abuse of exercise is a justification of this brief protest. The race for health is a corollary of the race for wealth, and in cities, where both forms of endeavour are liable to tax all our resources, the holiday season brings special risks in the over-anxiety to be strong.

